# The Clean Milk Campaign

**Published:** 1916-09-16

**Authors:** 


### THE CLEAN MILK CAMPAIGN.

The practical benefits which would have followed upon the Milk and Dairies Act of 1914 had, unfortunately, to be postponed until " such date, not being later than the expiration of one year after the termination of the present war, as the Local Government Board may by Order appoint." The National Clean Milk Society has, nevertheless, not suspended its activities. We appraise at a high value the efforts of this Society, the avowed aims of which are to raise the hygienic standard of milk and milk products and to educate the public as to the importance of a clean and wholesome milk supply, and we follow with interest and appreciation such popular articles as those which the Society has published in the *Observer*. Questions connected with milk supply have an especial importance in their relation to hospital work and sick dietary.

Realising, as the Society does, that the Local Government Board has power to issue " Milk and Dairies Orders " making various provisions, and in anticipation of such Orders, a Conference has been held to consider what their nature and scope should be. It is suggested that beneficial results will depend almost entirely on the terms of these Milk and Dairies Orders and upon their proper enforcement. The Report of the Conference has been forwarded to the President of the Local Government Board.

The resolutions of the Conference, which it is hoped will be taken into consideration by the Board in making Orders, cover much that is familiar to readers of The Hospital and to students of the many vexed questions of milk supply. Their aim is, of course, to prevent the sale of impure, stale, or dirty milk. It is proposed to authorise, control, and protect the sale of " certified milk," which means a milk of accepted quality and necessarily of higher price; such milk should be required to conform to a bacteriological standard: this method of controlling sale is reported to work well and has been extensively adopted in America. Other recommendations are with regard to the cooling of milk, the permissible amount of sediment, the " age " of milk at the time of sale, registration of dairies, inspection of premises and cows, and the sanitation of farms and dairies. A suggestion new to us is that satisfactory artificial lighting should be provided in cowsheds; it is certainly unlikely that many country farm cowsheds have at present any means of artificial lighting which could be termed satisfactory. An attempt to enforce the adoption of smaller churns for the conveyance of milk is proposed; we consider that the further suggestion that " the mouth of every churn should be completely covered when the churn is closed " needs amplification, as many churns now in use may be said to comply with this requirement, but are none the less uncleanly and dangerous. The Score Card System is recommended as being the best method of recording and comparing the results of inspection.

